# Expanding the Spectrum of Central Sensitivity Syndrome: Integrating Otologic Migraine as Otologic Central Sensitivity Syndrome

**DOI:** 10.3390/brainsci16030257

**Published:** 2026-02-25

**Authors:** Ghaidaa S. Khlaifat, Karen Tawk, Ella J. Lee, Khushi Bhatt, Mehdi Abouzari, Hamid R. Djalilian

**Affiliations:** 1 Department of Otolaryngology-Head and Neck Surgery, University of California, Irvine, CA 92612, USA; 2 Department of Biomedical Engineering, University of California, Irvine, CA 92612, USA; 3 Department of Neurosurgery, University of California, Irvine, CA 92612, USA

**Keywords:** central sensitivity syndrome, dizziness, hearing loss, migraine, tinnitus, vertigo

## Abstract

**Highlights:**

**What are the main findings?**

- Non-headache migraine manifestations—including tinnitus, hyperacusis, vertigo, dizziness, sudden hearing loss, and aural fullness—share core neurobiological features of central sensitization and are best conceptualized within a unified framework termed otologic central sensitivity syndrome (CSS).
- Reframing these otologic symptoms under the CSS paradigm provides a coherent explanation for their overlap, chronicity, and comorbidities, aligning clinical observations with neuroimaging and mechanistic evidence of central nervous system hyperexcitability.

**What are the implications of the main findings?**

- Adopting the otologic CSS framework may enable earlier recognition, reduce misdiagnosis and stigma (particularly when headache is absent), and support more targeted, mechanism-based management strategies.
- This conceptual shift encourages clinicians to move beyond headache-centric migraine definitions, validating otologic symptoms as centrally mediated and promoting more comprehensive, multidisciplinary care that can improve patient outcomes and quality of life.

**Abstract:**

**Objective**: To propose that migraine-related symptoms such as dizziness, sudden hearing loss, tinnitus, and vertigo—when occurring without headache—should be recognized as manifestations of central sensitivity syndrome (CSS), and to explore the implications of this reclassification for clinical practice and patient care. **Data sources**: PubMed Central and Google Scholar. **Review methods**: A search of the literature was performed using PubMed and Google Scholar. Search terms included combinations of keywords such as “migraine”, “vertigo”, “tinnitus”, “dizziness”, “sudden hearing loss”, “central sensitivity syndrome”, and “central sensitization”. **Conclusions**: Non-headache migraine symptoms show significant overlap with characteristics of CSS, including central nervous system hyperexcitability and dysregulation. Neuroimaging and clinical data support this connection, suggesting these symptoms may be better understood within the CSS framework. Recognizing this association could represent a conceptual shift in how such symptoms are classified and managed. **Implications for practice**: Incorporating non-headache migraine symptoms into the CSS paradigm may lead to earlier recognition, reduce misdiagnosis and stigma, and support the development of more effective, targeted therapeutic strategies for affected patients.

## 1. Introduction

Migraine is a multifaceted neurological disorder characterized by unilateral or sometimes bilateral pulsating headaches lasting 4 to 72 h [1,2]. The headache is often accompanied by autonomic symptoms, cognitive impairments, and sensory disturbances such as photophobia, phonophobia, muscle tenderness, and cutaneous allodynia [1]. Migraine headache is more common in women [3,4,5,6]. Pathophysiologically, migraine involves cortical spreading depression, trigemino-vascular activation, and altered sensory processing [7,8,9,10,11]. Beyond headache, migraine may present with a range of symptoms, including auditory and vestibular complaints such as dizziness, hyperacusis, tinnitus, and vertigo, are increasingly recognized as common components of migraine [12,13,14,15,16,17]. These otologic manifestations frequently occur independently of headache episodes, underscoring that migraine is far more than a headache, affecting multiple physiological and neurological domains. When considering the sheer number of areas affected, it would seem that migraine is often challenging to diagnose within the traditional migraine framework [13]. To address this clinical complexity, we have previously introduced the term “otologic migraine” to describe the spectrum of migraine-related vestibular, auditory, and auricular symptoms [18,19,20].

These atypical presentations aligns with the concept of acephalgic migraine, characterized by the presence of aura without headache [21,22,23]. Aura may include a range of neurological and visual symptoms, including hemianopsia, scotomata, amaurosis fugax, diplopia, tunnel vision, and other transient deficits [24,25]. While up to 44% of migraine patients with aura experience such auras without associated headaches [26], the condition often lacks familial patterns, with only 24% of patients reporting a family history [27]. This low family history percentage is likely due to poor recognition of atypical migraine symptoms in family members. Recurrent episodes of vertigo, frequently identified as having a migrainous origin, are often observed independently of any accompanying headache [28]. This broader spectrum of acephalgic migraine, in which periodic, non-headache symptoms such as vertigo or visual aura replace the typical headache presentation, highlights the heterogeneity of migraine presentations. Diagnosis remains reliant on clinical history, the exclusion of organic causes, and response to antimigraine therapies, further highlighting their shared pathophysiological basis [22]. Although the concept of migraine equivalents has existed for centuries, its recognition remains limited, and some question whether these symptoms are truly part of the migraine spectrum. In the absence of a definitive diagnostic test for atypical migraine, including otologic migraine, debate persists regarding whether these diverse symptoms arise from the same cortical spreading depression and neurovascular dysfunction underlying migraine pathology.

A compelling framework that might unify these varied clinical features is the concept of central sensitization (CS), a physiological mechanism characterized by dysregulation within the central nervous system (CNS), leading to neuronal hyperexcitability and altered sensory processing [29]. This phenomenon manifests as increased sensitivity to both noxious and non-noxious stimuli [30]. One presentation of CS is characterized by hyperalgesia, defined as an exaggerated sensitivity to stimuli that are normally less painful [29]. It also involves an expansion of the receptive field, wherein pain perception extends beyond the typical distribution of the affected peripheral nerve [31]. Additionally, CS is marked by an abnormally persistent pain that continues well beyond the cessation of the initial stimulus, often described as throbbing, burning, tingling, or numbness [32].

**Figure 1:**
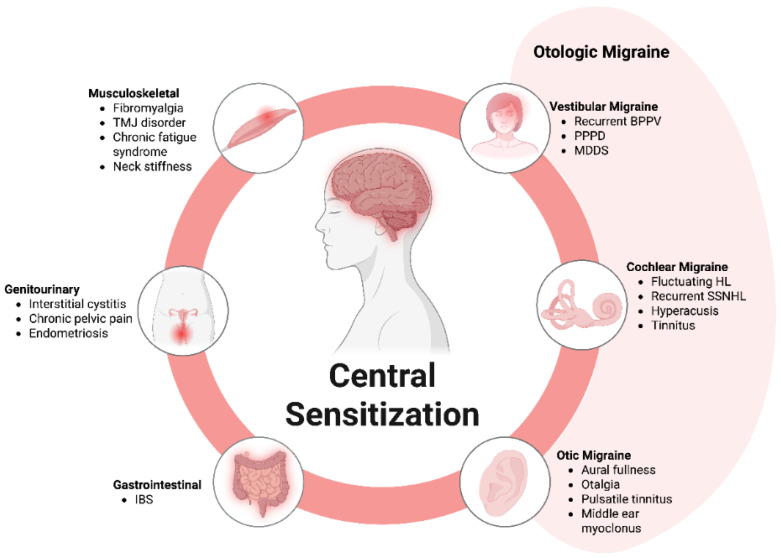
Central Sensitivity Syndromes. Central sensitization underlies central sensitivity syndromes, including fibromyalgia, irritable bowel syndrome, restless leg syndrome, post-traumatic stress disorder, vulvodynia, chronic fatigue syndrome, temporomandibular joint disorder, and migraine, highlighting shared pathophysiology and overlapping symptoms.

CS has been proposed as the underlying mechanism for “Central Sensitivity Syndromes” (CSSs) [33,34], a group of medically unexplained conditions lacking identifiable organic causes [33,34,35,36]. These syndromes include fibromyalgia (FM), chronic fatigue syndrome (CFS), multiple chemical sensitivity (MCS), temporomandibular joint disorder (TMD), and migraine [33,35,37,38,39,40,41,42] (Figure 1). Evidenced supporting CS involvement in migraine pathology includes impaired descending pain inhibitory control [43], its association with acute allodynia and the progression to headache chronification [44].

These CSSs are highly interconnected, sharing overlapping symptoms such as pain, and consistently demonstrate evidence of CS [33,35,37]. Interestingly, CS and its associations with CSSs are also observed in conditions where pain is not the predominant symptom, such as restless leg syndrome (RLS) [39,45,46] and post-traumatic stress disorder (PTSD) [39,47,48] (Figure 1). Beyond classic examples like FM, CFS, and MCS, disorders characterized by visceral hypersensitivity, including irritable bowel syndrome (IBS), chronic or interstitial cystitis, and vulvodynia, are also considered part of this spectrum [32,33,35,37,39]. Initially regarded as distinct nosological entities, these disorders have demonstrated significant overlap over time, with a high degree of co-occurrence [32,33,35,39,47,49]. This evidence supports a unified pathogenic network, termed CSS, encompassing these interrelated disorders [50,51].

The concept of neuronal sensitization, first proposed by Kandel [52] in 1976 and later expanded by Woolf [53], Yunus [32,54], Staud [55], and Nijs [56], serves as a unifying neurophysiological mechanism underlying CSS. It is characterized by an amplified and maladaptive neurosensory response to non-noxious stimuli, driven by heightened nociceptive synaptic transmission in receptors and neurotransmitters in the spinal cord and CNS. This results in increased neuronal excitability, impaired nociceptive inhibition, and a chronic state of neural hyperactivity. While initially developed to explain neuropathic pain [36,53,57,58], CS has since been extended to other pain conditions such as complex regional pain syndrome, chronic pelvic pain, TMD, and migraine [40,42,59,60,61,62]. Beyond pain, CS has been linked to abnormal responses to various stimuli, including neurocognitive or physical fatigue [55] and chemical or environmental agents, which provoke similar pathways [63,64,65,66]. The persistence of CS is strongly associated with the neuroinflammation phenomenon, in which pro-inflammatory mediators like TNF-α cross the blood–brain barrier, triggering a chronic cerebral autoinflammatory response [67]. Similarly, in conditions where a chronic inflammation could result in neural hyperactivity and, therefore, be a potential source of CS, it remains uncertain whether or not inflammation persists, as CS is one of the underlying neurophysiological mechanisms of nociplastic pain [68]. Notably, CSS demonstrates a pronounced female gender bias, likely due to estrogen’s role in potentiating brain sensitization [69,70,71,72,73].

Psychosocial factors play a role in the development and maintenance of CS [74,75], in which CS acts as a mediator between psychological factors and the intensity of the experienced pain [76]. For example, studies on chronic low back pain show that psychosocial factors—such as stress, trauma, depression, anxiety, maladaptive illness perceptions, and pain-related worrying—interact with this physiological sensitization, further amplifying symptom severity [77,78]. Additionally, prolonged psychological stress, PTSD, and histories of physical or psychological abuse further heighten susceptibility [48]. Individuals with childhood-onset PTSD, in particular, face a greater risk of developing CS, possibly due to brain developmental changes and the extended timeframe during which sensitization can evolve from childhood into adulthood [79,80,81,82,83,84]. Collectively, the interplay of genetic predisposition, environmental triggers (e.g., chemical exposure, infections, dietary changes), and psychosocial stressors creates a cascade leading to CS [63,64,65,66,85]. This process may begin as localized pain in a specific area that later persists, or hypersensitivity to similar triggers, but over time, it can expand to other systems (amplification) and contribute to the emergence of comorbid CSS conditions [86]. The resulting neurophysiological and functional burden—spanning physical, cognitive, and psychological domains—leads to significant impairment in daily and occupational activities and incurs substantial socioeconomic costs [39,87].

Evidence suggests that CS contributes to a variety of symptoms beyond pain [88,89], which explains why some patients report seemingly unrelated complaints. Prominent examples of central hypersensitivity include fibromyalgia and migraine, both of which exhibit symptomatology extending far beyond pain alone [49,89,90]. Given the broad and often overlapping symptom profile associated with CS and migraine, particularly involving otologic symptoms, there is a need for precise and meaningful clinical terminology to better capture this complexity.

**Hypothesis.**  *We propose that non-headache migraine symptoms in the ear (dizziness, hyperacusis, tinnitus, vertigo, aural fullness/pain, etc.) represent clinical expressions of CSS. This conceptualization aligns these symptoms with a broader spectrum of disorders characterized by CNS hyperexcitability and provides a unifying framework for understanding their pathophysiology and management.*

## 2. Review Methods

This study was conducted as a narrative literature review. Search terms included combinations of keywords such as “migraine”, “vertigo”, “tinnitus”, “dizziness”, “sudden hearing loss”, “central sensitivity syndrome”, and “central sensitization”. Searches were performed to identify publications addressing migraine, central sensitization, central sensitivity syndromes, and associated otologic manifestations. The search strategy encompassed publications from database inception through 2025 and included both foundational historical studies and contemporary research articles to provide conceptual and mechanistic context. Peer-reviewed original research articles, clinical studies, review articles, neuroimaging studies, and relevant theoretical papers published in English were considered. Articles were screened based on title and abstract for relevance to the proposed conceptual framework, and additional references were identified through manual review of reference lists from selected articles. Given the conceptual nature of this work, formal systematic review methodology (e.g., PRISMA framework) was not applied. A total of 260 citations were included.

## 3. Discussion

### 3.1. Association Between Central Sensitization and Migraine/Migraine-Related Symptoms

Within the framework of central sensitization, repeated exposure to specific stimuli—such as intense light, odors, and sound—can result in heightened sensitivity and the development of cross-sensitization [91,92,93]. Patients with migraine exhibit atypical brain activation in response to sensory input, especially involving the limbic system, visual cortex, and rostral pons [94,95,96]. Clinically, this heightened responsiveness presents as photophobia, phonophobia, osmophobia, and nausea—all of which correlate with migraine severity [97,98,99].

In the context of migraine pathophysiology, CS has been linked to activation of the trigeminal nucleus caudalis [10,44], a brainstem region that plays a pivotal role in orofacial pain and autonomic symptoms relevant to otolaryngology. For example, cranial parasympathetic symptoms—such as lacrimation, nasal blockage, and rhinorrhea—often seen in migraine patients, reflect dysregulated trigemino-autonomic activity sustained by CS [90,100]. Another significant aspect of pain sensitivity in migraine, similar to the hypersensitivity to light and sound experienced by patients, is hypersensitivity to touch, clinically described as cutaneous allodynia and hyperalgesia [101,102]. Allodynia refers to pain elicited by normally non-painful stimuli and often results in referred pain that often extends beyond the head and face to other body regions during migraine episodes [61,103]. Patients with severe allodynia were found to have a longer history of migraine and were more likely to exhibit symptoms of anxiety, depression, and smoking behavior [104]. Bernstein and colleagues [61,103] observed that cutaneous allodynia developed in 79% of migraine patients during attacks, often extending beyond the area of referred pain, a finding that has been consistently corroborated in subsequent studies [104,105,106,107,108]. Furthermore, spontaneous body pain and allodynia have been reported as prodromal symptoms preceding migraine episodes [103,109,110]. Fibromyalgia and migraine are the most widely accepted examples of CSS, with both conditions producing a range of symptoms beyond pain alone [88,89]. This multi-symptom presentation may result from CS, which explains why patients often report seemingly unrelated symptoms.

CS is a multifaceted process influenced by various mechanisms, including synaptic plasticity driven by neuroinflammation and the action of cytokines [111]. Burstein et al. [112] propose two mechanisms by which hypothalamic and brainstem neurons can initiate headaches. First, hypothalamic neurons may trigger meningeal nociceptor activation by increasing parasympathetic tone [113,114], stimulating the superior salivatory nucleus [115,116] and releasing vasodilatory and inflammatory mediators [117,118,119]. This enhanced parasympathetic activity, seen during migraine episodes, can be alleviated by sphenopalatine ganglion blockade [113,120,121,122,123,124,125]. Second, hypothalamic and brainstem neurons, which regulate responses to deviations in physiological and emotional homeostasis, play a role in lowering the threshold for the transmission of nociceptive trigemino-vascular signals by releasing neuromodulators such as dopamine, histamine, orexin, melanin-concentrating hormone, norepinephrine and serotonin [126,127], thereby influencing the allostatic load—the brain’s stress-regulating set point—which may explain the circadian and trigger-specific nature of migraine attacks [126,127,128,129].

**Figure 2:**
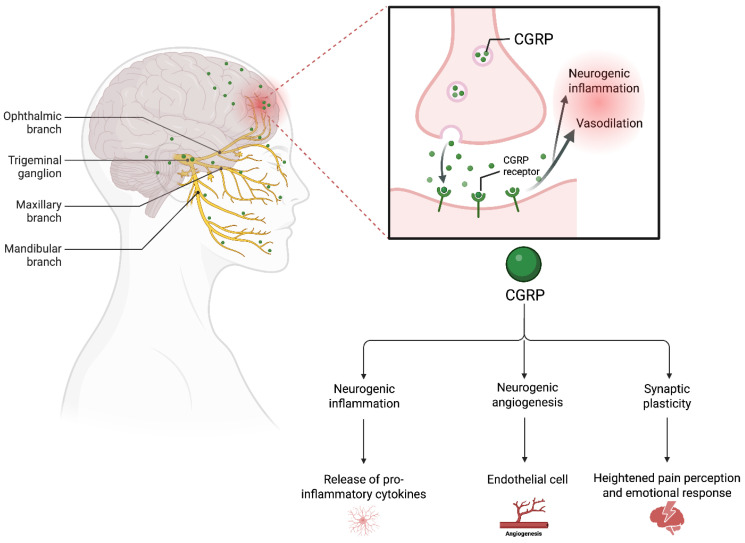
CGRP Role In Central Sensitization. Calcitonin Gene-Related Peptide drives migraine by promoting vasodilation of cerebral and meningeal blood vessels, neurogenic inflammation, and sustained nociceptive signaling after trigemino-vascular activation, reinforcing its central role in sensitization and headache.

Key molecular pathways involved in sensitization include the up-regulation of glutamate receptors and activation of protein kinase C-mediated phosphorylation of transient receptor potential (TRP) channels, particularly transient receptor potential vanilloid 1 (TRPV1), by inflammatory mediators [130,131,132]. These TRP channels have become significant targets in drug research aimed at mitigating pain sensitization [133,134]. Calcitonin gene-related peptide (CGRP), highly expressed in trigeminal neurons and released peripherally, contributes to central sensitization and heightened pain signaling (Figure 2) [135]. CGRP-related pathways, combined with reduced pain inhibition and the activation of NMDA receptors, create a hyperexcitable state in the central nervous system [136]. This hyperexcitability underpins persistent chronic pain and abnormal sensory experiences such as allodynia and hyperalgesia [136]. Importantly, while pain is the hallmark symptom of central sensitization, it is not the only manifestation. This underscores the complex nature of central sensitization and its involvement in a broad spectrum of sensory and neurological dysfunctions. In addition to allodynia and hyperalgesia, symptoms such as dysosmia, dysgeusia, hyperacusis, and dizziness are thought to result from nerve dysfunction without detectable structural abnormalities. Although the exact mechanisms remain elusive, evidence points to disruptions in dopamine, serotonin, and norepinephrine metabolism as key contributors [137]. This observation highlights the potential therapeutic value of pharmacological interventions targeting these neurotransmitter systems for alleviating some of these symptoms. The overlap between central and peripheral sensitization further explains why allodynia and hyperalgesia frequently coexist, highlighting the interconnected and multifaceted nature of sensitization processes [138].

### 3.2. Functional and Structural Changes in the Migraineurs

Research has revealed significant functional and structural changes in the brains of individuals with migraine. Functional neuroimaging studies have demonstrated increased activity in various brain regions, such as the periaqueductal gray, red nucleus, substantia nigra, hypothalamus, posterior thalamus, cerebellum, insula, cingulate cortex, prefrontal cortex, hippocampus, and anterior temporal pole [139,140,141,142,143,144,145,146], while decreased activation occurs in regions such as the somatosensory cortex, nucleus cuneiformis, caudate, putamen, and pallidum [145,146,147,148,149,150]. These alterations, particularly in the cingulate and prefrontal cortex, appear to occur in response to repetitive stimuli [145,146,147,148,149,150]. Evidence from studies examining auditory and nociceptive processing suggests that the migraine brain demonstrated an impaired ability to habituate to repetitive stimuli [151]. For example, in passive “oddball” auditory tasks, where individuals hear a sequence of repetitive sounds interrupted by rare, deviant tones, migraineurs exhibit amplified neural responses to these rare tones over time, in contrast to the decreasing responses typically seen in non-migraineurs. This potentiation reflects a lack of habituation, indicating abnormal sensory filtering and heightened cortical responsiveness in migraine [152]. Similarly, interictal deficits in habituation, as measured by the nociceptive blink reflex, further support this observation [153]. These findings have contributed to the hypothesis that the migraine brain is characterized by an exaggerated response to sensory inputs, distinct from the concept of hyperexcitability [151,152,153,154,155,156].

Advanced imaging techniques, such as voxel-based morphometry and diffusion tensor imaging, have revealed significant structural differences in the brains of migraine patients compared to controls. These studies demonstrate thickening of the somatosensory cortex and increased gray matter volume in the caudate nuclei, particularly in individuals with high-frequency migraine [157]. Conversely, reduced gray matter has been observed in pain-processing regions, including the anterior cingulate cortex, amygdala, insula, operculum, and various frontal, temporal, and precentral gyri. These structural changes, which are linked to alterations in pain pathways, appear to vary based on the frequency and intensity of migraine attacks [8,148,157,158,159,160]. Similar patterns of brain remodeling are observed in other chronic pain conditions, such as chronic low back pain, chronic pelvic pain, and osteoarthritis, suggesting shared mechanisms underlying chronic pain syndromes [161,162,163].

### 3.3. Otologic Migraine and Migraine-Related Symptoms Share Similarities with CSS-Related Disorders

The Central Sensitization Inventory (CSI) is a validated self-administered questionnaire designed to assess symptoms associated with CS and screen for related conditions, including FM, CFS, MFS, RLS, and chronic headaches [86,164]. Migraine and its otologic-associated symptoms (e.g., loud or fluctuating tinnitus, hearing loss, vertigo, and dizziness) share significant overlaps with CSS-related disorders. For example, hyperacusis experienced by migraine patients may parallel the sound hypersensitivity observed in conditions like FM and temporomandibular disorders. Similarly, migraine and IBS share commonalities in comorbidities and potential pathophysiological mechanisms, including pain characteristics and treatment responses, suggesting they may exist on a spectrum of central sensitization-related disorders [165]. Neuroimaging studies provide further insights, revealing impaired inhibitory control in the anterior cingulate cortex and hyperactivation of the insula and amygdala as potential drivers of central sensitization in disorders like fibromyalgia and IBS [166,167]. Interestingly, these activation patterns appear consistent across various pain syndromes and CSS-related conditions, raising questions about whether differences between migraine-related and somatic pain arise from distinct central pain-processing mechanisms or reflect shared pathways [161,162]. Beyond the hallmark symptoms—headache in migraine, widespread pain in FM, and persistent fatigue in CFS—patients with these conditions often exhibit otolaryngologic symptoms such as loud or fluctuating tinnitus, hearing loss, ear fullness/pressure, otalgia, and hyperacusis [168,169,170]. Despite their prevalence, these manifestations are not traditionally included in diagnostic criteria and are thus frequently underrecognized. The heightened sensory processing characteristic of CS may underlie the emergence of these otologic symptoms across these conditions.

### 3.4. Tinnitus

Tinnitus, defined as the perception of sound without an external source, is closely linked to stress, anxiety, and depression, with studies showing that psychological factors can exacerbate tinnitus symptoms [171,172,173,174]. Studies indicate that heightened emotional states such as frustration, grief, and stress, particularly during events like the COVID-19 lockdown, can exacerbate tinnitus symptoms [175]. Tinnitus is generated in the central auditory pathways and caused by a loss of hearing cells or synaptopathy in the cochlea [171,176]. Tinnitus is modulated by an atypical migraine process which causes more distress due to the loudness [177]. Primary chronic tinnitus (PCT), often accompanied by hearing loss seen on the audiogram, appears to involve a central mechanism akin to central sensitization. PCT frequently persists or worsens even after severing cochlear input to the brain, resembling the central mechanisms of sensitization observed in FM and other chronic pain syndromes or phantom limb pain [178]. While peripheral damage initially triggers tinnitus, its persistence seems to be mediated by central neural mechanisms, further reinforcing its classification as a centrally driven condition [178].

Recent studies have explored the potential link between migraine and inner ear disorders, such as tinnitus, sensorineural hearing loss, and vertigo [13,14,17,18,19,177,179,180,181,182,183,184,185,186,187,188,189,190]. Vestibular migraine and cochlear migraine are particularly associated with inner ear dysfunction [13,182,191,192,193]. Vestibular migraine, characterized by a combination of migraine and vertigo symptoms, has been linked to higher rates of tinnitus in patients [12,193,194,195,196,197,198,199,200]. In contrast, cochlear migraine involves migraine symptoms without vestibular effects but with notable cochlear symptoms [184,192]. Tinnitus, results from damage to the peripheral auditory system, is believed to stem from damage to hair cells in the cochlea, particularly the organ of Corti [201]. This damage leads to reduced lateral inhibition and reorganization of the auditory cortex, triggering spontaneous neuronal activity perceived as tinnitus [201]. An active migraine process heightens central sensitivity, leading to exacerbation of tinnitus [177,196]. Trigeminal nerve activation during migraine episodes can lead to neurogenic inflammation and altered cochlear blood flow, contributing to hearing loss and tinnitus perception as well, which are termed transient ear noises or fleeting tinnitus [202,203,204]. Studies have shown that migraine patients exhibit reduced otoacoustic emissions, indicating cochlear dysfunction. Additionally, vascular changes in the inner ear, influenced by trigeminal nerve activity, likely play a role in the onset of tinnitus during migraine attacks [184,205].

Acute tinnitus associated with migraine is thought to arise from central sensitization, where distorted auditory signals during central compensation are perceived as tinnitus [206]. This theory is supported by findings that patients with migraine report intensified tinnitus during migraine attacks [207]. Tinnitus is also particularly common among individuals with FM, with studies reporting a higher prevalence of self-reported tinnitus in FM patients compared to the general population [208,209]. The onset of tinnitus in these patients often follows the development of FM, likely driven by shared central sensitization processes [210,211]. Pharmacologic interventions, including anticonvulsants and antidepressants, have shown potential in alleviating tinnitus severity in FM, underscoring the role of central mechanisms in its persistence [186,212,213,214].

### 3.5. Hearing Loss

Research has established a compelling association between migraine and sudden sensorineural hearing loss (SSNHL), with studies indicating that migraineurs have a significantly higher risk of developing SSNHL compared to non-migraineurs [180,190,192,215,216,217,218,219]. A large population-based cohort study found that the incidence of SSNHL in migraine patients was nearly double that of matched controls, with an incidence rate of 81.6 per 100,000 person-years compared to 45.7 in controls [180]. While the exact mechanisms remain unclear, case reports and histopathological studies suggest that vascular dysfunction, such as cochlear vasospasm and ischemic damage, may contribute to the condition [220,221,222,223,224]. For example, postmortem findings in a patient with migraine and SSNHL revealed ischemic fibrosis in the cochlea, implicating vascular compromise in its pathology [220].

Beyond vascular factors, emerging hypotheses suggest that inflammation and cellular stress pathways—including those involving nuclear factor-kappa B, CGRP, and substance P—may also play a role in the pathogenesis of SSNHL in migraineurs [206,225,226]. Studies have identified potential links between migraine-associated inflammation and cochlear dysfunction, with inflammatory mediator genes implicated in SSNHL [227]. Additionally, research on vestibular migraine highlighted a spectrum of auditory symptoms, including mild and reversible hearing loss, high-frequency deficits, and tinnitus, which appear to progress over time [198,199,200,228,229]. Preventive migraine therapies, such as nortriptyline, topiramate, and intratympanic steroid injections, have shown promise in improving auditory outcomes in patients with SSNHL, underscoring the importance of considering migraine as a potential underlying factor in SSNHL [18,186,230]. These findings call for further investigation into the shared pathophysiological mechanisms of migraine and SSNHL to inform early intervention and targeted treatments.

Recent studies have also explored the relationship between FM and hearing loss [231]. For example, Le et al. reported an increased risk of sensorineural, conductive, and mixed hearing loss in FM patients [232]. Koca et al. observed higher hearing thresholds across all frequencies in FM patients compared to controls, despite no prior history of hearing impairment [210].

Additionally, tinnitus and hearing loss have been frequently reported in FM patients [208,233]. In one study, 16.7% of FM patients experienced tinnitus, while 12.5% reported hearing loss [208]. These findings suggest a multifactorial link between FM and auditory dysfunction. Given the overlap in mechanisms such as inflammation and altered sensory processing observed in both FM and migraine-related auditory disorders, further investigation is warranted to clarify the shared pathways contributing to hearing loss in these conditions.

### 3.6. Vertigo and Dizziness

Studies have consistently shown a strong association between migraine and vertigo, with evidence indicating that vertigo is more prevalent among individuals with migraine and vice versa [184,234,235,236,237,238,239]. This combination has been described using various terms, such as migraine-associated vertigo, migraine-associated dizziness, and migrainous vertigo [235,240,241,242]. Vestibular migraine (VM), a term introduced by Dieterich and Brandt, refers to vestibular symptoms causally linked to migraine and is now widely accepted in clinical neurology [15]. Recently, the International Classification of Headache Disorders (ICHD) and the International Bárány Society, established diagnostic criteria for VM [21,243]. It is worth noting that the Bárány Society’s International Classification of Vestibular Disorders distinguishes dizziness, defined as spatial disorientation, from vertigo, described as an illusion of motion, although vestibular symptoms have historically been referred to by both terms [244]. VM is considered the most common cause of recurrent spontaneous vertigo and accounts for a significant proportion of patients seen in both dizziness and migraine clinics, yet it remains underdiagnosed [16,235,245]. Epidemiological studies reveal that VM occurs more frequently in women [235,246,247,248,249]. While vertigo can precede, accompany, or follow a headache, it may also manifest as isolated vertigo episodes, particularly in postmenopausal women [250]. VM can develop at any age but typically affects individuals with a history of migraine, often with an average diagnostic delay of 8.4 years [251,252,253,254,255]. The underlying mechanisms connecting migraine and vestibular symptoms are rooted in the convergence of vestibular and cranial nociceptive pathways [256,257]. Experimental studies highlight shared neurochemical properties between trigeminal and vestibular ganglion cells, including serotonin and capsaicin receptors, which converge in brainstem structures such as the raphe nuclei, parabrachial nucleus, and locus coeruleus [258,259,260]. These areas not only modulate pain sensitivity but also contribute to anxiety responses, potentially explaining the co-occurrence of migraine, balance disorders, and anxiety [261].

Functional imaging studies further highlight the overlap between vestibular and pain pathways at the brainstem, thalamic, and cortical levels, including the cingulate gyrus, orbitofrontal cortex, and insula [262,263,264]. For instance, increased metabolism in temporoparietal-insular areas and bilateral thalamus has been observed during vestibular migraine attacks, while gray matter reductions in regions associated with pain and vestibular processing suggest a pathoanatomic link between the two systems [264,265]. This interplay of pathways underscores the hyperexcitability of the vestibular system in individuals with migraine, providing insight into their shared pathophysiology and complex clinical presentations.

## 4. Implications for Practice

Migraine and related central sensitivity syndromes encompass a range of sensory disturbances beyond headache, notably including otologic symptoms such as tinnitus, hearing loss, vertigo, and hyperacusis. These symptoms share common neurobiological mechanisms rooted in central sensitization, supporting their classification under a broader framework we propose as an “otologic central sensitivity syndrome.” This terminology liberates clinicians and patients from the limitations of the word “migraine,” which is often narrowly interpreted as headache alone. By shifting the focus to central sensitization, clinicians can validate and treat a wider range of sensory symptoms without needing to justify a diagnosis of migraine in the absence of headache. Ultimately, this approach may enable more comprehensive patient care and improved patient quality of life.

## 5. Conclusions

Conceptualizing certain non-headache migraine symptoms within the CSS framework may provide a unifying hypothesis for understanding shared neurobiological mechanisms underlying symptoms such as dizziness, hearing loss, loud or fluctuating tinnitus, and vertigo. While this model offers a potentially useful lens through which to interpret overlapping clinical phenotypes, current evidence remains largely associative and inferential. Further prospective studies integrating clinical phenotyping, mechanistic biomarkers, and longitudinal outcomes are necessary to determine whether central sensitization meaningfully contributes to those otologic presentations and whether this framework improves diagnostic clarity or therapeutic decision-making. At present, the central sensitization paradigm should be viewed as a hypothesis-generating model rather than a validated reclassification of these conditions.

## Data Availability

No new data were created or analyzed in this study. Data sharing is not applicable to this article.
